# Phase I trial of ATM inhibitor M3541 in combination with palliative radiotherapy in patients with solid tumors

**DOI:** 10.1007/s10637-022-01216-8

**Published:** 2022-02-12

**Authors:** Saiama N. Waqar, Clifford Robinson, Anthony J. Olszanski, Alexander Spira, Melissa Hackmaster, Luisa Lucas, Laura Sponton, Hulin Jin, Ursula Hering, Damien Cronier, Marianna Grinberg, Annick Seithel-Keuth, Ivan Diaz-Padilla, Jordan Berlin

**Affiliations:** 1 Division of Oncology, Washington University School of Medicine and Alvin J. Siteman Cancer Center, Saint Louis, MO USA; 2 Department of Radiation Oncology, Washington University School of Medicine, Saint Louis, MO USA; 3 Department of Hematology/Oncology, Fox Chase Cancer Center, Philadelphia, PA USA; 4 Medical Oncology, Virginia Cancer Specialists Research Institute and US Oncology Research, Fairfax, VA USA; 5 Merck S.L.U., Mollet del Valles, Spain, an affiliate of Merck KGaA, Darmstadt, Germany; 6 The Healthcare Business of Merck KGaA, Darmstadt, Germany; 7 Oncology Global Clinical Development, Ares Trading SA, Eysins, Switzerland, an affiliate of Merck KGaA, Darmstadt, Germany; 8 Division of Hematology/Oncology, Vanderbilt-Ingram Cancer Center, Nashville, TN USA

**Keywords:** M3541, ATM inhibitor, Solid tumor, Palliative radiotherapy, DNA damage response, DNA repair

## Abstract

*Background*. Ataxia telangiectasia mutated (ATM) kinase orchestrates DNA double strand break (DSB) repair; ATM inhibitors may therefore enhance the therapeutic effect of DSB-inducing treatments such as radiotherapy (RT). M3541 is an orally administered selective inhibitor of ATM. *Methods.* This phase I dose-escalation study evaluated the maximum-tolerated dose (MTD), recommended phase II dose(s) (RP2D), safety, pharmacokinetics (PK) and antitumor activity of M3541 in combination with fractionated palliative RT in patients with solid tumors. Fifteen patients received palliative RT (30 Gy in 10 fractions) and escalating doses of M3541 (50–300 mg administered on RT fraction days) guided by a Bayesian 2-parameter logistic regression model with overdose control. *Results.* Doses of M3541 up to 300 mg/fraction day were well tolerated. One patient (200 mg group) experienced two dose-limiting toxicities (urinary tract infection, febrile neutropenia) that resolved with antibiotics. All patients reported ≥ 1 treatment-emergent adverse event (TEAE) but none led to treatment discontinuation. No grade ≥ 4 TEAEs were reported and there was no indication of a dose effect for any TEAE. Three patients (20.0%; 95% confidence interval 4.3–48.1) had confirmed complete or partial response. M3541 total plasma levels did not increase with dose following single or repeated dosing. No relationship was observed between dose and changes in the ratio of phosphorylated to total ATM or in immune cell counts. *Conclusions.* The MTD and RP2D could not be established as the study closed early due to the absence of a dose–response relationship and non-optimal PK profile. No further clinical development of M3541 was pursued. (*Trial registration number* ClinicalTrials.gov NCT03225105. Registration date July 21, 2017).

**Supplementary Information:**

The online version contains supplementary material available at 10.1007/s10637-022-01216-8.

## Introduction

More than 50% of patients with cancer receive radiotherapy (RT) during the course of their disease, of which approximately half are estimated to receive palliative RT [1]. A key therapeutic mechanism of RT is via the production of DNA double strand breaks (DSB), similar to other DNA-damaging agents [2]. Complex molecular networks, known as the DNA damage response, have evolved to repair such breaks and maintain the integrity of the genome [3, 4]. A major regulator in the DNA damage response is ataxia telangiectasia mutated (ATM) kinase. ATM helps to maintain genome integrity in healthy cells by orchestrating the repair of DSBs and by acting as an upstream signaling kinase, regulating cell-cycle checkpoint control in the response to DSB damage [2, 5].

When double-stranded DNA is damaged, ATM is activated through autophosphorylation, the first step in a finely tuned signaling network of effector proteins and substrates controlled by ATM, and including checkpoint kinase 2 (CHK2) [6, 7]. Upon phosphorylation by ATM, CHK2 kinase activity in turn modulates further reactions, inducing cell-cycle arrest [2, 4]. Inhibitors of ATM kinase are therefore expected to suppress DSB DNA repair, block checkpoint controls and enhance the therapeutic effect of RT and other DSB-inducing agents [8]. However, due to a high degree of homology of the kinase domain of ATM and other phosphatidylinositol 3-kinase-related kinases (PI3Ks), off-target inhibiton is important to recognize, and selective ATM inhibitors are needed [8].

M3541 was designed to be an orally administered, sub-nanomolar inhibitor of ATM. M3541 was shown to be a highly potent inhibitor of ATM (IC_50_ < 1 nM) and also highly selective, with an IC_50_ of > 100 nM for 99.3% of 292 investigated human kinases [9]. In particular, M3541 showed negligible inhibition against the closely related kinase family members ataxia telangiectasia and Rad3-related (ATR), DNA-dependent protein kinase (DNA-PK), PIK3 isoforms, and mammalian target of rapamycin (mTOR) [9]. Preclinical experiments showed that M3541 sensitizes tumor cell lines to radiation therapy in vitro and strongly enhances the antitumor activity of ionizing radiation in vivo in preclinical models [9, 10]. These effects are due to the inhibition of ATM kinase activity, as demonstrated by reduced phosphorylation levels of CHK2, in human tumor cell lines. It was therefore hypothesized that treatment of oligo-metastatic disease with M3541 plus RT would be more effective than RT alone. Furthermore, it was hypothesized that M3541 could be used for palliation of problematic lesions or as a bridging therapy between systemic treatments.

We report the results of a phase I trial (NCT03225105) of M3541 in combination with palliative RT in patients with solid tumors.

## Methods

### Patients

Male or female patients aged ≥ 18 years and having solid tumors with malignant lesions in the thorax, abdominal cavity, head and neck region, or extremities (any histology) likely to benefit from palliative RT were eligible. Other key inclusion criteria were: Eastern Cooperative Oncology Group performance status (ECOG PS) ≤ 2; life expectancy ≥﻿ 3 months; adequate hematologic, hepatic, and renal function; and agreement to use highly effective contraception.

Patients requiring palliative RT for lesions in the spine or lesions adjacent to the spinal cord were excluded from this study. Further exclusion criteria were the use of other anticancer therapies within 5 × their elimination half-life, or the use of any investigational agent within 28 days, before the first dose of M3541, and residual toxicity due to previous anticancer therapy. Patients at increased risk for radiation toxicities, such as known collagen vascular disease, were also excluded.

The study was conducted in accordance with the ethical principles of the International Council for Harmonisation (ICH) guidelines for Good Clinical Practice and the Declaration of Helsinki, and was approved by the relevant Ethics Committees. All screened patients signed an informed consent form.

### Study design and treatment plan

This phase I, open-label, multicenter, dose-escalation study was designed to evaluate the safety, tolerability, pharmacokinetics (PK), pharmacodynamics (PD), and antitumor activity of orally administered M3541 in combination with fractionated palliative RT in patients with solid tumors.

To provide an orally administered drug, M3541 was incorporated in a polymer matrix using cellulose acetate phthalate to enhance bioavailability, and then formulated and coated to provide an immediate-release tablet.

All patients received RT over 2 consecutive calendar weeks (Monday through Friday, with the intervening Saturday and Sunday as M3541/RT holidays). The total RT dose was 30 Gy given in 10 fractions of 3 Gy/fraction day (FD) on FD 1 to FD 10. On M3541 treatment days, M3541 was administered 105 min ± 60 min before RT. Criteria were specified for dose modification of either or both interventions if toxicities occurred.

The dose-limiting toxicity (DLT) evaluation period consisted of the scheduled 2-week RT treatment period plus a 3-week DLT follow-up period for the first three cohorts. The protocol was then amended to specify a follow-up DLT period of 2 weeks for subsequent cohorts, to allow patients to receive subsequent anticancer therapy if they experienced rapid disease progression (see Supplement for details of allowed treatment).

The starting dose for the M3541 once per FD schedule was 50 mg orally/FD. After all patients in a cohort had been treated and completed the DLT evaluation period, the Safety Monitoring Committee (SMC) guided dose escalation using a Bayesian 2-parameter logistic regression model with overdose control. Up to eight cohorts were planned with data from three patients per dose level. The biologically effective daily dose in humans was estimated, based on a tumor static concentration of 82.5 mg/kg in mice (corresponding to an area under the concentration–time curve [AUC] of 1,208 μg*h/mL), to range from 150 to 500 mg QD on a 6-week cycle (5 days on/2 days off).The preplanned dose levels were 50 mg, 100 mg, 200 mg, 300 mg, 500 mg, and 800 mg orally once per FD.

### Objectives

The primary objective was to determine the maximum tolerated dose and recommended phase II dose(s) (RP2D) for M3541 in combination with fractionated palliative RT. Secondary objectives were to evaluate the safety profile and tolerability, the antitumor activity, and the PK of M3541 in combination with fractionated palliative RT. Exploratory objectives included treatment-related changes in PD markers and the impact on the immune system of M3541 in combination with fractionated palliative RT.

### Key endpoints

The primary safety endpoint was the occurrence of DLTs during the DLT evaluation period (see Supplement for definition of DLTs.) Secondary safety endpoints were treatment-emergent adverse events (TEAEs), grade ≥ 3 adverse events (AEs), serious adverse events (SAEs), deaths, and laboratory evaluations assessed from the first administration of M3541 onwards. All AEs were coded using the Medical Dictionary for Regulatory Activities (MedDRA) Version 22.1 and were graded according to the National Cancer Institute (NCI) Common Terminology Criteria for Adverse Events CTCAE Version 4.03 [11].

Efficacy endpoints included best overall response (BOR), determined using Response Evaluation Criteria in Solid Tumors version 1.1 (RECIST 1.1) [12] as assessed by the investigator every 6 weeks (starting on post-treatment day 42) for the first 6 months, and every 12 weeks thereafter, until evidence of disease progression. Tumor responses were derived from the sum of diameters of target lesions, non-target lesions and new lesions; target lesions included both irradiated and non-irradiated lesions.

### Assessment of PK and biomarkers

PK endpoints included calculation of PK parameters from blood samples collected at predefined timepoints pre-dose, after first drug administration (FD 1) and multiple dose administrations (FD 9 or FD 10).

The PD biomarker of ATM inhibition by M3541 is the ratio of phosphorylated and total forms of the ATM protein, the proximal target of the ATM self-phosphorylating activity, as measured in serial peripheral blood mononuclear cells (PBMC) isolated from whole blood samples collected at baseline (before treatment) and during treatment.

All PBMC samples were stimulated ex vivo with the DNA double strand break inducer bleomycin, which activates DNA damage repair by ATM autophosphorylation. MesoScale Discovery-based electrochemiluminescence-based assays were validated and used to measure the phosphorylated ATM (pATM) and total ATM (tATM) concentrations in samples from all participants. The pATM/tATM ratio and changes from bleomycin-stimulated baseline over time were calculated.

Immunophenotyping analyses were conducted to assess the effects of the M3541 treatment in combination with RT on circulating immune cells, mainly as a distal PD biomarker [13]. In this biomarker analysis, baseline (FD1) and after treatment (FD6, FD9) whole blood samples were collected from each individual participant to monitor the changes in immune cell count and percentage of subset types of T cells, B cells, monocytes and natural killer cells.

### Statistical analyses

Data cutoff for the primary analyses was defined by the completion of the safety follow-up period (30 days post-treatment) of the last patient in the last cohort.

Analysis sets were defined as follows: safety analysis, all patients who received at least one dose of M3541; DLT set, all patients who received at least 80% of the M3541 and RT planned dose and completed the DLT evaluation period, or received at least one administration of M3541 and experienced a DLT during the DLT evaluation period; PK analysis set, all patients who received at least one dose of M3541 and provided at least one quantifiable post-dose concentration; biomarker analysis set, all patients who received at least one dose of M3541 and provided a quantifiable blood sample for biomarker analysis both pre- and post-treatment with M3541. In this study, the safety, DLT and PK analysis sets included all patients.

The BOR was defined as the best response assessed for target lesions, non-target lesions and new lesions across all time points evaluated per RECIST 1.1 using the investigator-reported overall response per time point and excluding assessments after tumor surgery. The objective response rate (ORR) was defined as the proportion of patients with BOR of complete response (CR) or partial response (PR). Assessments of CR and PR had to be confirmed at least 4 weeks after the initial response assessment. To be considered the best response, stable disease had to have met the minimum 6 weeks (with an allowed visit window of –7 days) from first day of treatment.

The safety endpoints were tabulated by dose level using descriptive statistics. Summary statistics were used for the efficacy endpoints by dose level (safety analysis set). The 2-sided exact Clopper﻿–Pearson 95% confidence interval (CI) was used for the ORR. Biomarker data for the biomarker analysis set were summarized descriptively by dose level and time point.

## Results

### Patient characteristics and disposition

The study was conducted at five centers between September 2017 and April 2020, with the last treatment administered in September 2019.

A total of 21 patients were screened for participation in the study. Six patients did not start treatment (four did not meet eligibility criteria, one withdrew consent, one decided to pursue a different study), and 15 (100.0%) received study treatment across four M3541 dose cohorts, all of whom completed their course of treatment. At the study data cutoff date (September 16, 2019), seven patients (46.7%) had discontinued from the study, due to death (*n* = 6; 40.0%) or withdrawn consent (*n* = 1; 6.7%). Eight patients (53.3%) were continuing in the study in follow-up.

Patient characteristics and sites of irradiation are shown in Table 1. Most patients were white (*n* = 11; 73.3%) and approximately half were female (*n* = 8; 53.3%), with a median age of 62 years. Baseline ECOG PS was 0 for three patients (20.0%) and 1 for 12 patients (80.0%). Cancer types included lung (*n* = 6; 40.0%), colorectal (*n* = 2; 13.3%), sarcoma (*n* = 2; 13.3%) bladder, eccrine porocarcinoma, liver, mesothelioma, and pancreatic (all *n* = 1; 6.7%). One patient (6.7%) had Stage III disease at study entry, all other patients (*n* = 14; 93.3%) had Stage IV disease.

**Table 1:** Patient demographics and baseline characteristics (*N* = 15; safety analysis set). Values are *n* (%) unless stated otherwise

Characteristic	Value
Sex	
Male	7 (46.7)
Female	8 (53.3)
Ethnicity	
White	11 (73.3)
Black or African American	4 (26.7)
Age [median (range), years]	62 (41–76)
Weight [median (range), kg]	74.1 (47.4–135.5)
ECOG PS at baseline	
0	3 (20.0)
1	12 (80.0)
Cancer types	
Lung	6 (40.0)
Colorectal	2 (13.3)
Sarcoma	2 (13.3)
Other	5 (33.3)
Location of irradiated areas	Thorax/lung (*n* = 5) Thorax/thoracic wall (*n* = 2) Abdomen/abdomen wall Abdomen/liver Abdomen/pancreas Abdomen/perirectal mass/peritoneal anterior pelvis/peritoneal Abdomen/pelvic node Head and neck nodes Bone/thoracic spine

*ECOG PS* Eastern Cooperative Oncology Group performance status

There were no differences across the dose cohorts in demographics or baseline characteristics that might be expected to influence the results.

### Safety

Fourteen of 15 patients (93.3%) received 10 days of study treatment across 10 FD; one patient (6.7%) in the 300 mg/FD cohort received 11 M3541 doses over 19 days (this patient received one M3541 dose without RT due to technical issues leading to a delay in radiation initiation, 1 week prior to FD1). All patients received ≥ 80% of their intended M3541 dose and all patients received 100% of the intended RT dose (30 Gy for all the M3541 dose cohorts).

Doses of M3541 up to 300 mg/FD were well tolerated. There were no clinically relevant late radiotoxicity effects observed in the follow up period of one year. One patient (6.7%) in the 200 mg cohort experienced two DLTs. This 76-year-old male had Stage IV bladder cancer and study treatment included RT to lymph nodes in the pelvis. On study day 14, 2 days after the last study treatment, he was hospitalized with a grade 3 urinary tract infection (UTI) and grade 3 febrile neutropenia. The grade 3 UTI was unrelated to M3541 and RT. The grade 3 febrile neutropenia was related to M3541 but unrelated to RT. The patient was treated with antibiotics and both events resolved within 1 week.

With regard to SAEs, across all four M3541 dose cohorts, two patients were reported with treatment-emergent SAEs: one patient in the 100 mg/FD dose cohort and one patient in the 200 mg/FD dose cohort (already mentioned above). In both cases the SAE was a UTI considered unrelated to RT or M3541. There were no M3541-related SAEs. The 76-year-old patient already described above had two RT-related SAEs of increased blood creatinine and dehydration.

With regard to TEAEs, all patients across all four M3541 dose levels reported at least one TEAE: there were ten patients (66.7%) with M3541-related TEAEs and ten patients with RT-related TEAEs (Table 2a). The TEAEs at the Preferred Term level reported for ≥ 3 patients (≥ 20%) overall in descending order were diarrhea and fatigue (each five patients, 33.3%), pruritus (four patients, 26.7%), and dry skin, muscular weakness, nausea, oropharyngeal pain, and UTI (each three patients, 20.0%) (Table 2b).

**Table 2:** TEAEs for M3541 + RT (*N* = 15; safety analysis set). **a** Overview of TEAEs. **b** Incidence of TEAEs by System Organ Class and Preferred Term reported for ≥ three patients (20%) overall at the Preferred Term Level (*N* = 15; safety analysis set)

Patients with any: *n* (%)	Any grade				Grade ≥ 3^a^
TEAE	15 (100.0)				4 (26.7)
M3541-related TEAE	10 (66.7)				1 (6.7)
RT-related TEAE	10 (66.7)				2 (13.3)
Serious TEAE	2 (13.3)				2 (13.3)
M3541-related serious TEAE	0				0
RT-related serious TEAE	1 (6.7)				1 (6.7)
TEAE leading to study discontinuation	0				0
TEAE leading to death	0				0
	**M3541 dose cohort**				
**System Organ Class Preferred Term**^**b**^	**50 mg/FD** ***n* = 3** ***n* (%)**	**100 mg/FD** ***n* = 4** ***n* (%)**	**200 mg/FD** ***n* = 5** **n (%)**	**300 mg/FD** ***n* = 3** **n (%)**	**Overall** ***N* = 15** **n (%)**
Patients with ≥ 1 TEAE^c^	3 (100.0)	4 (100.0)	5 (100.0)	3 (100.0)	15 (100.0)
Gastrointestinal disorders	3 (100.0)	1 (25.0)	3 (60.0)	1 (33.3)	8 (53.3)
Diarrhea	0	1 (25.0)	3 (60.0)	1 (33.3)	5 (33.3)
Nausea	1 (33.3)	0	2 (40.0)	0	3 (20.0)
General disorders and administration site conditions	3 (100.0)	3 (75.0)	2 (40.0)	1 (33.3)	9 (60.0)
Fatigue	2 (66.7)	0	2 (40.0)	1 (33.3)	5 (33.3)
Infections and infestations	1 (33.3)	1 (25.0)	2 (40.0)	0	4 (26.7)
UTI	0	1 (25.0)	2 (40.0)	0	3 (20.0)
Musculoskeletal and connective tissue disorders	2 (66.7)	3 (75.0)	2 (40.0)	1 (33.3)	8 (53.3)
Muscular weakness	0	2 (50.0)	1 (20.0)	0	3 (20.0)
Respiratory, thoracic and mediastinal disorders	1 (33.3)	1 (25.0)	1 (20.0)	1 (33.3)	4 (26.7)
Oropharyngeal pain	1 (33.3)	1 (25.0)	1 (20.0)	0	3 (20.0)
Skin and subcutaneous tissue disorders	0	2 (50.0)	3 (60.0)	2 (66.7)	7 (46.7)
Dry skin	0	1 (25.0)	1 (20.0)	1 (33.3)	3 (20.0)
Pruritus	0	1 (25.0)	1 (20.0)	2 (66.7)	4 (26.7)

*FD, fraction day, RT* radiotherapy, *MedDRA* Medical Dictionary for Regulatory Activities, *N/n* number of patients, *TEAE* treatment-emergent adverse event, *UTI* urinary tract infection

^a^No grade 4 TEAEs were reported

^b^All TEAEs were coded using the MedDRA Version 22.1

^c^If a patient had more than one event within a primary System Organ Class or Preferred Term, the patient was counted only once within that System Organ Class or Preferred Term

No TEAEs of grade 4 or grade 5 were reported and no patients discontinued treatment or the study due to a TEAE. No TEAEs of lymphopenia or decreased lymphocyte count were reported. Laboratory findings showed transient, mainly mild decreases in lymphocyte counts, with a nadir close to the end of the treatment period.

The maximum tolerated dose according to the Bayesian model could not be determined due to early termination of the study.

### Preliminary efficacy

The BOR was confirmed PR for three (20.0%) patients with lung cancer (50 mg, *n* = 1; 200 mg, *n* = 2). Irradiated lesions in these patients were located in the lung (*n* = 2) and pelvic lymph node (*n* = 1), and non-irradiated lesions were located in the lymph nodes (*n* = 2), adrenal gland (*n* = 1) and lung (*n* = 1). In all three patients, a size decrease in 1–2 non-irradiated lesions was observed, in addition to an irradiated lesion. Nine (60.0%) patients had a BOR of SD (50 mg, *n* = 1; 100 mg, *n* = 2; 200 mg, *n* = 3; 300 mg, *n* = 3). Three (20.0%) patients had progressive disease at the irradiation site. A waterfall plot of the best percentage change in tumor size from baseline is shown in Supplementary Fig. 1. The ORR for confirmed BOR was three patients (20.0%; 95% CI 4.3–48.1).

### Pharmacokinetics

**Fig. 1:**
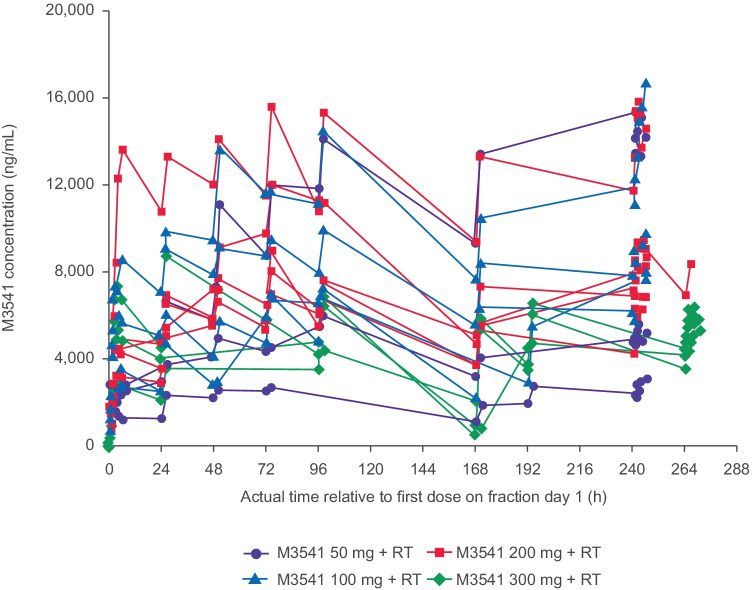
Individual M3541 plasma concentration–time profiles by dose level for planned fraction days 1 to 10 (*n* = 15; PK analysis set)

M3541 total plasma levels increased less than dose-proportionally (50–300 mg) following single or repeated dosing (Fig. 1, Table 3). Exposure ([AUC_0–24_, h*ng/mL] and maximum observed plasma concentration [C_max_]) increased between the 50 mg and 100 mg doses after both single and multiple dosing, but not at higher doses (Table 3). The rate of elimination, as judged by the decline between 96 h (FD5) and 168 h (FD6), appeared consistent across all dose groups (Fig. 1).

**Table 3:** PK parameters of M3541 after **a** single-dose administration and **b** multiple-dose administration, shown as geometric mean (geometric coefficient of variation, %)

Dose (mg)	*N*	C_max_ (ng/mL)	C_max_/dose (ng/mL/mg)	AUC_0-24_ (h*ng/mL)	AUC_0-24_/dose (h*ng/mL/mg)	AUC_0-∞_ (h*ng/mL)	AUC_extra_% (%)
50	3	2550 (43.0)	50.9 (43.0)	50,000 (48.6)	1000 (48.6)	229,000 (NC) [1]*	87.8 (NC) [1]*
100	4	4750 (53.2)	47.5 (53.2)	96,400 (54.2)	964 (54.2)	546,000 (NC) [1]*	89.9(NC) [1]*
200	5	5350 (59.4)	26.8 (59.4)	110,000 (54.9)	551 (54.9)	551,000 (NC) [1]*	88.2 (NC) [1]*
300	3	4850 (49.9)	16.2 (49.9)	90,900 (45.1)	303 (45.1)	NC	NC
**Dose (mg)**	***N***	**C**_**max**_ **(ng/mL)**	**C**_**max**_**/dose** **(ng/mL/mg)**	**AUC**_**0-6**_ (h*ng/mL)	**AUC**_**0-6**_**/dose** (h*ng/mL/mg)		
50	3	6420 (96.7)	128 (96.7)	34,900 (102)	698 (102)		
100	4	10,500 (32.6)	105 (32.6)	56,600 (30.6)	566 (30.6)		
200	5	9660 (30.9)	48.3 (30.9)	53,100 (30.9)	266 (30.9)		
300	3	5870 (8.02)	19.6 (8.02)	32,000 (8.19)	107 (8.19)		

*AUC* area under the curve, *AUC*_*0-24*_ AUC over 24 h, *AUC*_*0-∞*_ AUC from dosing time extrapolated to infinity, *AUC*_*extra*_*%* AUC from time last extrapolated to infinity given as percentage of AUC0_0-∞,_ *AUC*_*0-6*_ AUC over 6 h, *C*_*max*_ maximum concentration, *N* number of patients, *NC* not calculated

[*N*]* represents a parameter-specific *N* value

### Biomarker

Engagement of M3541 with its target, as assessed by a decrease from baseline after ex vivo stimulation in the ratio pATM/tATM over time in PBMCs, was observed after 24 h in most participants’ samples. While a trend towards a decrease in the PD parameter with increasing concentration was observed, the relationship with exposure to M3541 could not be reliably established due to high biological variability of the PD parameter and the small sample size (*n* = 14) (Supplementary Fig. 2).

**Fig. 2:**
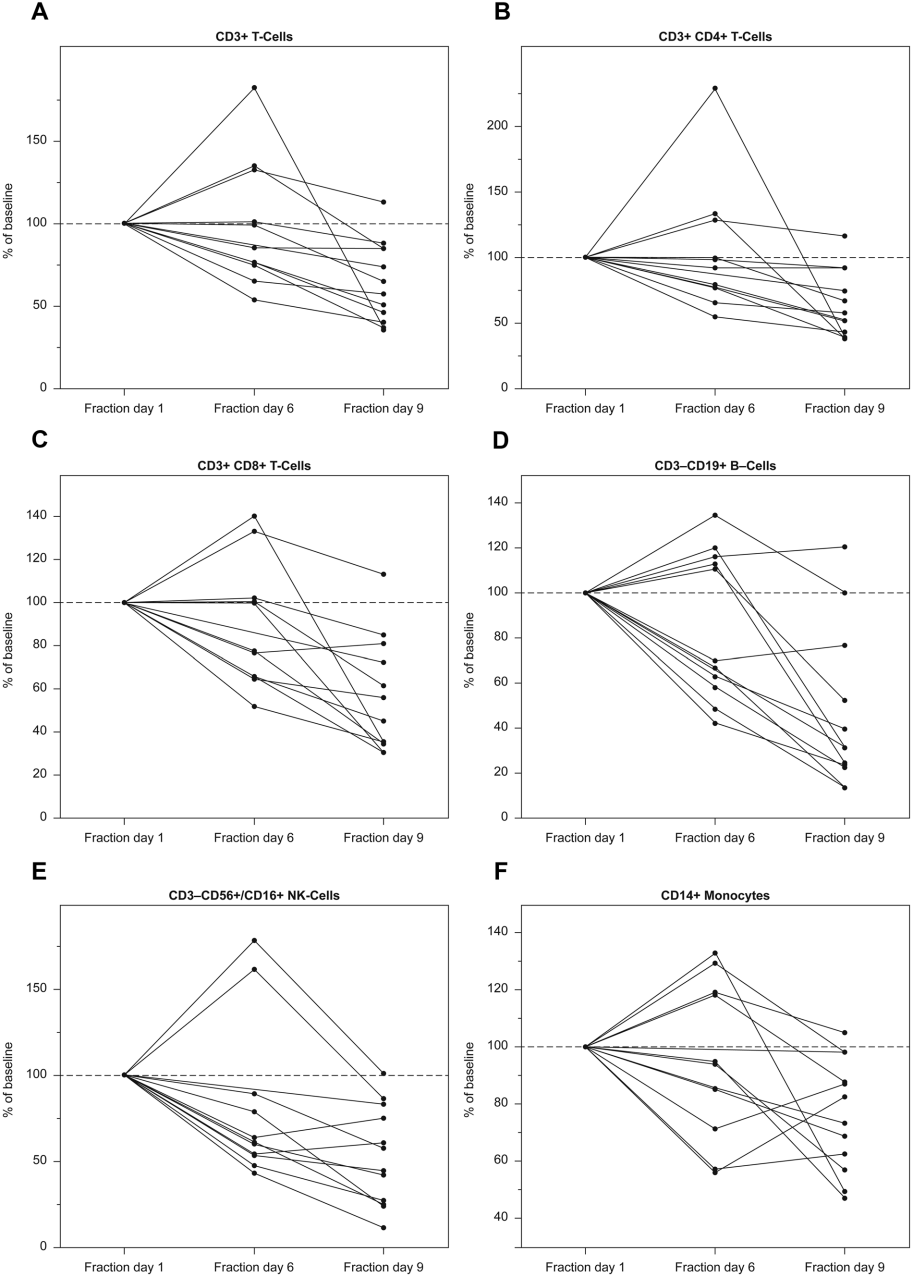
Changes in immune cell counts from fraction day 1 (FD1; set as 100%) to FD6 and FD9 for: **A** total CD3 + T cells, **B** CD4 + T cells, **C** CD8 + T cells, **D** CD19 + B cells, **E** CD3–CD56 + /CD16 + NK cells, **F** CD14 + monocytes in whole blood over time (*n* = 12; biomarker analysis set with complete FD1, FD6, FD9 patients only). Note different y-axis scales

The cell counts and percentage relative to baseline of immune cell types in whole blood decreased over time during the treatment, in particular for CD8 + T-cell, B-cell and natural killer cell subsets (Fig. 2). The reductions were not associated with M3541 dose.

## Discussion

To our knowledge this is the first clinical study reporting the use of an ATM inhibitor (M3541) in combination with palliative RT in patients with advanced solid tumors. Prior preclinical results had shown that M3541 sensitized multiple tumor cell lines to ionizing radiation therapy in vitro [9, 10]. Furthermore, oral administration of M3541 to nude mice bearing human tumor xenografts in a clinically relevant radiation regimen led to complete tumor regression [10]. However, the results from the current phase I study in humans indicated no dose dependency for the PK and PD of M3541.

M3541 had a manageable toxicity profile in combination with low-dose palliative RT (30 Gy), with no significant safety concerns. Observed TEAEs at doses ranging from 50 to 300 mg were as expected based on preclinical toxicology data observed in nonclinical studies (e.g., gastrointestinal side effects, hematopoietic and lymphatic system effects with infections) and some early radiotoxicity to the skin. DLTs were reported for only one patient. There was no indication of any dose effect for any TEAE, possibly because exposure did not increase across dose levels.

Based on the non-clinical safety profile of M3541, lymphatic system toxicity with the risk of lymphocytopenia was considered an important potential risk. In the current study, no TEAEs of lymphopenia or lymphocyte count decreased were reported, and only transient, mainly mild decreases in lymphocyte counts were observed, despite reductions in cell counts and percentage relative to baseline of immune cell subsets. There was no relationship between these reductions and M3541 dose.

M3541 plasma levels increased in a less than dose proportional manner over the dose range of 50 mg to 300 mg following single or repeated dosing. While the achieved exposure exceeded the predicted exposure and the predicted efficacious plasma concentration, activity at these plasma levels was limited and exposure did not increase further with dose. Since the rate of elimination appeared consistent across all dose groups, this finding points towards a limitation in the fraction absorbed. Animal studies showed rapid absorption of M3541 in rat and dog, with a median time to maximum concentration of 1–2 h. In rats, the increase in area under the concentration–time curve (AUC) was roughly dose-proportional from 10 to 75 mg/kg, whereas the maximum observed plasma concentration (C_max_) was less so. In dogs, both AUC and C_max_ increased roughly dose-proportionally for doses up to 10 mg/kg/day, but not at 30 mg/kg/day (data on file). Although the formulation of M3541 had been chosen to facilitate absorption and bioavailability in humans, the physicochemical properties of the drug did not allow a greater fraction to be absorbed at doses above 100 mg in humans. Hence, no further clinical development of M3541 was pursued. Despite efforts to investigate the absorption determinants of M3541, there is insufficient evidence to determine the main driver for the limited absorption observed in this study.

In line with the PK results, the biomarker (pATM/tATM and lymphocyte subsets) concentration–time profiles showed no apparent dose dependency. M3541 treatment in combination with RT induced a reduction in T cells, B cells, NK cells and monocytes in several patients. Again, the trend in this change was not associated with dose levels. These changes may have resulted from the RT, which is known to reduce circulating immune cells [14]. However, it cannot be excluded that the observed effect was induced by M3541 or M3541 in combination with RT.

The lack of correlation between the administered dose and exposure, as well as the lack of a clear PD signal, may partially explain the lack of a clear efficacy signal. An objective response was observed in just three patients (20.0%; 95% CI 4.3–48.1), which could well have corresponded to the single effect of the RT. In those patients with tumor shrinkage, there were no additional clinical findings (such as prolonged disease control or notable sustained symptom improvement) that may have suggested an enhancement of efficacy by the addition of M3541 to palliative radiation.

Research has shown that some cancers have *ATM* gene mutations which render them more sensitive to anticancer therapy, and that this can be exploited by using targeted therapies which induce a synthetic lethal status. [2, 15] In parallel, the use of drugs that inhibit the repair of DNA DSBs, such as ATM inhibitors and DNA-dependent protein kinase inhibitors (e.g., peposertib [16]), has been considered a promising strategy to enhance the antitumor activity of DSB-inducing treatment modalities even in patients who have tumors without mutations. However, the DDR involves several overlapping pathways, so that the inhibition of any one element (such as ATM) may be compensated for by other effector/mediator proteins, and a better understanding of these pathways is needed [15]. Despite these challenges, it is of interest to keep investigating ATM inhibitors in conjunction with RT, given that RT is widely used and that efficacy improvements can be expected. M3541 had PK limitations that prevented an analysis of efficacy. Therefore, although the clinical development of M3541 was halted, the ATM pathway still represents an attractive therapeutic target. Indeed, the development of the second-generation ATM inhibitor M4076 is ongoing, [17] and the compound has entered clinical investigation (ClinicalTrials.gov NCT04882917).

## Supplementary Information

Below is the link to the electronic supplementary material.

Supplementary file1 (PDF 76 KB)

## Data Availability

Any requests for data by qualified scientific and medical researchers for legitimate research purposes will be subject to the healthcare business of Merck KGaA’s Data Sharing Policy. All requests should be submitted in writing to the healthcare business of Merck KGaA’s data sharing portal (https://www.merckgroup.com/en/research/our-approach-to-research-and-development/healthcare/clinical-trials/commitment-responsible-data-sharing.html). When the healthcare business of Merck KGaA has a co-research, co-development, or co-marketing or co-promotion agreement, or when the product has been out-licensed, the responsibility for disclosure might be dependent on the agreement between parties. Under these circumstances, the healthcare business of Merck KGaA will endeavor to gain agreement to share data in response to requests.
