# Author Correction: Phase I trial of ATM inhibitor M3541 in combination with palliative radiotherapy in patients with solid tumors

**DOI:** 10.1007/s10637-022-01226-6

**Published:** 2022-02-28

**Authors:** Saiama N. Waqar, Clifford Robinson, Anthony J. Olszanski, Alexander Spira, Melissa Hackmaster, Luisa Lucas, Laura Sponton, Hulin Jin, Ursula Hering, Damien Cronier, Marianna Grinberg, Annick Seithel-Keuth, Ivan Diaz-Padilla, Jordan Berlin

**Affiliations:** 1 Division of Oncology, Washington University School of Medicine and Alvin J. Siteman Cancer Center, Saint Louis, MO USA; 2 Department of Radiation Oncology, Washington University School of Medicine, Saint Louis, MO USA; 3 Department of Hematology/Oncology, Fox Chase Cancer Center, Philadelphia, PA USA; 4 Medical Oncology, Virginia Cancer Specialists Research Institute and US Oncology Research, Fairfax, VA USA; 5 Merck S.L.U, Mollet del Valles, Spain, an affiliate of Merck KGaA, Darmstadt, Germany; 6 The Healthcare Business of Merck KGaA, Darmstadt, Germany; 7 Oncology Global Clinical Development, Ares Trading SA, Eysins, Switzerland, an affiliate of Merck KGaA, Darmstadt, Germany; 8 Division of Hematology/Oncology, Vanderbilt-Ingram Cancer Center, Nashville, TN USA

## Investigational New Drugs https://doi.org/10.1007/s10637-022–01216-8

The article Phase I trial of ATM inhibitor M3541 in combination with palliative radiotherapy in patients with solid tumors was originally published electronically on the publisher’s internet portal on 12 January 2022 without open access. The author decided to opt for Open Choice and to make the article an Open Access publication. Therefore, the copyright of the article has been changed to © The Author(s) 2022 and the article is forthwith distributed under a Creative Commons Attribution 4.0 International License (https://creativecommons.org/licenses/by/4.0/), which permits use, sharing, adaptation, distribution and reproduction in any medium or format, as long as you give appropriate credit to the original author(s) and the source, provide a link to the Creative Commons licence, and indicate if changes were made.

The original article has been corrected.

