# Supplementary material for: Phase I trial of ATM inhibitor M3541 in combination with palliative radiotherapy in patients with solid tumors
Source: Invest New Drugs. 2022 Feb 12;40(3):596–605. doi: 10.1007/s10637-022-01216-8 (PMC9098584; doi:10.1007/s10637-022-01216-8)

PHASE I TRIAL OF ATM INHIBITOR M3541 IN COMBINATION WITH PALLIATIVE RADIOTHERAPY IN PATIENTS WITH SOLID TUMORS

Saiama N Waqar, Clifford Robinson, Anthony J Olszanski, Alexander Spira, Melissa Hackmaster, Luisa Lucas, Laura Sponton, Hulin Jin, Ursula Hering, Damien Cronier, Marianna Grinberg, Annick Seithel-Keuth, Ivan Diaz-Padilla, Jordan Berlin

SUPPLEMENTARY INFORMATION

METHODS

Allowed treatment during the study

The dose-limiting toxicity (DLT) evaluation period consisted of the scheduled 2-week radiotherapy treatment period plus a 3-week DLT follow-up period for the first three cohorts (12 patients in total). The protocol was then amended to specify a follow-up DLT period of 2 weeks for subsequent cohorts, to allow patients to receive subsequent anticancer therapy if they experienced rapid disease progression.

Any medications (other than those excluded below) necessary for the patients' welfare and not interfering with the study treatment could be given at the Investigator's discretion.

Allowed treatments were as follows:

- Systemic corticosteroids (<7 days)
- Anti-infectious drugs and hematopoietic growth factors, if medically indicated
- Patients with functional neuroendocrine tumor who progressed on octreotide or lanreotide could remain on octreotide or lanreotide for control of hormonal syndromes
- Patients under stable hormone therapy could remain on hormone therapy

The following treatments were prohibited:

- Chemotherapy, immunotherapy, biologic therapy, or any other anticancer therapy during the treatment and DLT period
- Radiotherapy (involving < 30% of bone marrow) to any other lesion was allowed at any time beyond post-treatment day 7

Definition of dose-limiting toxicities (DLTs)

A DLT was defined as any of the following adverse events (AEs) that occurred during the DLT evaluation period (except those that were clearly and incontrovertibly due to disease progression or extraneous causes), with severity graded according to the National Cancer Institute (NCI) Common Terminology Criteria for Adverse Events, Version 4.03 (CTCAE v4.03):

- Grade ≥ 3 non-hematologic toxicity, except:
    - Nausea, vomiting, and/or diarrhea lasting ≤ 3 days in the once per fraction day (FD) schedule that can be medically managed
    - Worsening of pre-existing tumor pain associated with tumor lesions for which the patient was irradiated in the context of this study
- Evidence of treatment-related hepatocellular injury for > 3 days in the once per FD schedule, such as ≥ 5 × the upper limit of normal (ULN) of alanine aminotransferase (ALT) or aspartate aminotransferase (AST) and without any other apparent clinical causality
- Any occurrence of Hy's law (defined as aminotransferases > 3 × ULN, alkaline phosphatase > 2 × ULN, total bilirubin ≥ 2 × ULN, with no other reason to account for these abnormalities)
- Febrile neutropenia or grade 4 neutropenia lasting for > 5 days, grade 4 thrombocytopenia or any requirement for platelet transfusion (as defined by grade 4 thrombocytopenia lasting for > 5 days or grade ≥ 3 thrombocytopenia with bleeding), grade 4 anemia that was unexplained by the underlying disease
- Any toxicity related to study treatment that caused the patient to interrupt treatment (defined as having to delay the next scheduled M3541 administration) for ≥ 4 FDs in the once per FD dosing schedule
- A related treatment-emergent adverse event (TEAE) that in the opinion of the Safety Monitoring Committee was of potential clinical significance such that further dose escalation would expose patients to unacceptable risk.

Supplementary Fig. 1 Waterfall plot of best percentage change in tumor size from baseline (*n* = 15; safety analysis set)

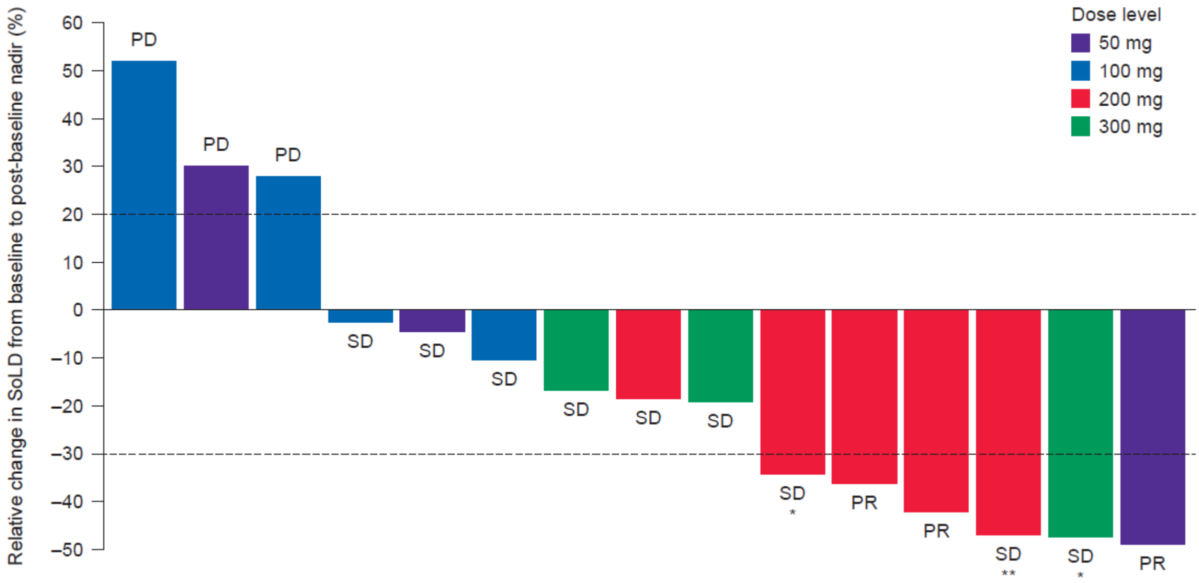

*Initial assessment of PR, subsequent assessment was missing, RECIST 1.1 classification[1] of SD.

**Initial assessment of PR, subsequent assessment was SD, RECIST 1.1 classification[1] of SD.

PD, progressive disease; PR, partial response; RECIST 1.1, response evaluation criteria in solid tumors version 1.1; SD, stable disease; SoLD, sum of lesion diameters (RECIST 1.1) [1].

Complete response (CR): Disappearance of all target lesions. Any pathological lymph nodes (whether target or non-target) must have reduction in short axis to < 10 mm.

Partial response (PR): At least a 30% decrease in the sum of diameters of target lesions, taking as reference the baseline sum diameters.

Progressive disease (PD): At least a 20% increase in the sum of diameters of target lesions, taking as reference the smallest sum on study (this includes the baseline sum if that is the smallest on study). In addition to the relative increase of 20%, the sum must also demonstrate an absolute increase of at least 5 mm. (Note: the appearance of one or more new lesions is also considered progression).

Stable disease (SD): Neither sufficient shrinkage to qualify for PR nor sufficient increase to qualify for PD, taking as reference the smallest sum diameters while on study.

Reference

1. Eisenhauer EA, Therasse P, Bogaerts J, et al. New response evaluation criteria in solid tumours: revised RECIST guideline (version 1.1). *Eur J Cancer.* 2009;45(2):228-247.

Supplementary Fig. 2 M3541 inhibition of ATM, assessed via an expected decrease in the pATM/tATM ratio versus concentration of M3541 up to 24 hours after the first drug administration (*n* = 14; biomarker analysis set).

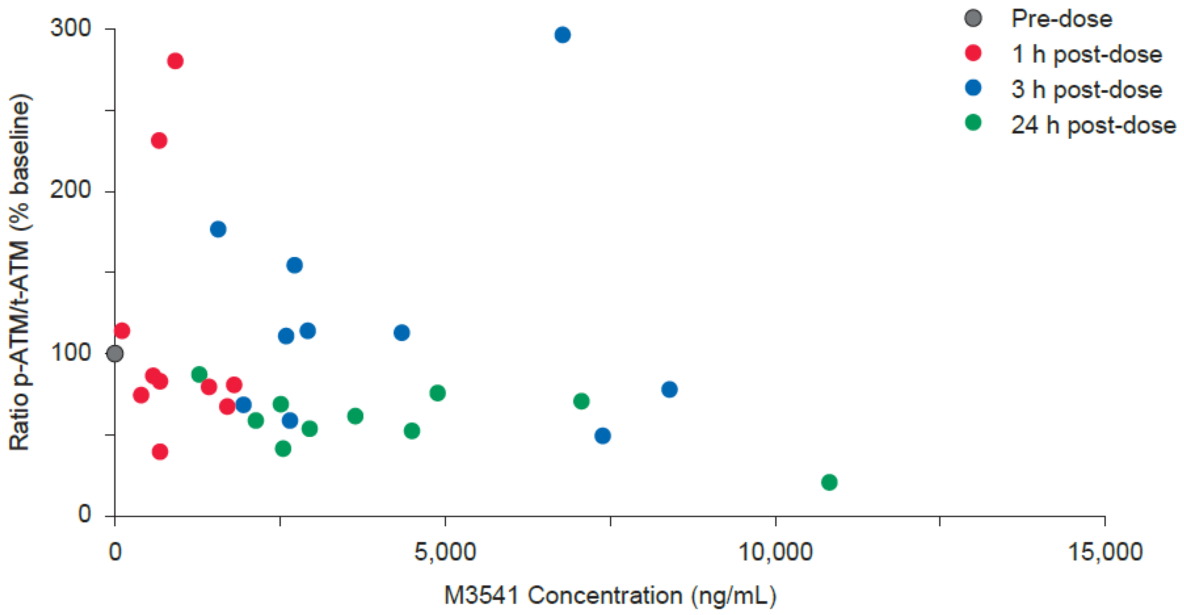

Supplement: Supplementary file 1 — Supplementary file1 (PDF 76 KB) [file 10637_2022_1216_MOESM1_ESM.pdf]
